# Optimal Experience and Personal Growth: Flow and the Consolidation of Place Identity

**DOI:** 10.3389/fpsyg.2016.01654

**Published:** 2016-11-07

**Authors:** Marino Bonaiuto, Yanhui Mao, Scott Roberts, Anastasia Psalti, Silvia Ariccio, Uberta Ganucci Cancellieri, Mihaly Csikszentmihalyi

**Affiliations:** ^1^Dipartimento di Psicologia dei Processi di Sviluppo e Socializzazione, Sapienza Università di RomaRoma, Italy; ^2^Centro Interuniversitario di Ricerca in Psicologia Ambientale, Sapienza Università di RomaRoma, Italy; ^3^School of Economics and Management, Southwest Jiaotong UniversityChengdu, China; ^4^Department of Psychology, Claremont Graduate University, ClaremontCA, USA; ^5^Department of Early Childhood Education, Alexander Technological Educational Institute of ThessalonikiThessaloniki, Greece; ^6^Università per Stranieri “Dante Alighieri” di Reggio CalabriaReggio Calabria, Italy

**Keywords:** eudaimonistic identity theory, flow experience, place identity, eudaimonia

## Abstract

This study examined the relationship between flow experience and place identity, based on eudaimonistic identity theory (EIT) which prioritizes self-defining activities as important for an individual’s identification of his/her goals, values, beliefs, and interests corresponding to one’s own identity development or enhancement. This study focuses on place identity, the identity’s features relating to a person’s relation with her/his place. The study is also based on flow theory, according to which some salient features of an activity experience are important for happiness and well-being. Questionnaire surveys on Italian and Greek residents focused on their perceived flow and place identity in relation to their own specific local place experiences. The overall findings revealed that flow experience occurring in one’s own preferred place is widely reported as resulting from a range of self-defining activities, irrespective of gender or age, and it is positively and significantly associated with one’s own place identity. Such findings provide the first quantitative evidence about the link between flow experienced during meaningfully located self-defining activities and identity experienced at the place level, similarly to the corresponding personal and social levels that had been previously already empirically tested. Results are also discussed in terms of their implications for EIT’s understanding and enrichment, especially by its generalization from the traditional, personal identity level up to that of place identity. More generally, this study has implications for maintaining or enhancing one’s own place identity, and therefore people–place relations, by means of facilitating a person’s flow experience within psychologically meaningful places.

## Introduction

### Flow, the Psychology of Optimal Experience through Various Activities

Optimal experience, or flow, within positive psychology, has received worldwide attention since its birth (Csikszentmihalyi, 1975/2000): it depicts the psychological mental state of a person who is immersed in an activity with energized concentration, optimal enjoyment, full involvement, and intrinsic interests, and who is usually focused, motivated, positive, energized, and aligned with the task at hand (Csikszentmihalyi, 1975/2000, 1990). The term “flow” describes optimal experiences that are among the most enjoyable in human life (Csikszentmihalyi, 1982), and such experience may emerge in any situation or place in which there is an ongoing activity (Csikszentmihalyi, 1990, 1997), as well as when there are clear goals, immediate feedback, and good balance between skills of a person and the challenge of the activity (Csikszentmihalyi and Csikszentmihalyi, 1988; Csikszentmihalyi and LeFevre, 1989; Waterman et al., 2003). It is therefore clear because fundamental to its very definition, the experience of flow has an intrinsic relation with the situation, and particularly with the interplay between personal characteristics and the features of the contextual surround: provided that a balance and match is realized between high individual’s skills and high contextual challenges. Indeed, a wide range of activities that are associated with flow refer to distinct settings and activities, like work (Csikszentmihalyi and LeFevre, 1989; Csikszentmihalyi and Kleiber, 1991; Demerouti, 2006; Nielsen and Cleal, 2010); education/study (Craig et al., 2004); sports (Jackson and Marsh, 1996; Kowal and Fortier, 1999; Harmison, 2006); combat activities in military and police (Nakamura and Csikszentmihalyi, 2002; Harari, 2008); music and arts (Csikszentmihalyi and Csikszentmihalyi, 1988; Csikszentmihalyi and LeFevre, 1989; Tietze, 2008); chess playing, rock climbing, and dance (Csikszentmihalyi, 1975/2000; online activities (Skadberg and Kimmel, 2004); digital games (Hamari et al., 2016), and so on. Various engaging activities are reported by a wide range of individuals: “*flow is reported in essentially the same words by men and women, by young people and old, by teenagers from Japan, by adults from India and Thailand, by old women from Korea, by Navajo shepherds, by farmers in Italy, and by workers on the assembly line in Chicago* (Csikszentmihalyi, 1990, p. 4)". This informative and descriptive finding seems to suggest that flow is broadly reported beyond gender, age, country, or culture. The empirical evidence of gender/age difference on flow has not yet been fully empirically tested, thus the present work considers gender and age difference on flow experience as a secondary aim.

### Place Identity, the Subjective Experience via Various Place Located Activities

Place identity (Proshansky, 1978; Proshansky and Fabian, 1987), as one of the prominent concepts within environmental psychology, has received worldwide attention mainly by environmental psychologists (e.g., Bonaiuto and Bonnes, 2000; Bonnes and Bonaiuto, 2002; Twigger-Ross et al., 2003). It is defined as “memories, conceptions, interpretations, ideas, and related feelings toward specific physical environments as well as types of settings” (Proshansky et al., 1983, p. 60), and the “physical world socialization of the self” (Proshansky et al., 1983, p. 57). As a facet of self-identity comparable to personal identity, place identity is considered as another aspect of one’s identity describing individuals’ identification with their physical world, and it is thus viewed as a sub-identity similar to other sub-levels such as personal identity and social identity (Twigger-Ross et al., 2003). It is based on the fact that the individuals’ positive self-esteem and emerging identity can be triggered from their physical environmental surroundings through various daily activities, in which the individuals interact with that place. The physical environment is established as an important factor for place identity, and indirectly for the whole identity of a person, since the meaningful place may provide an environmental and situational context for daily activities, facilitate human behavior and, as part of an individual’s range of places, could be incorporated into one’s self-concept. In further, most of the environmental psychology literature focusing on place identity addressed place identity by investigating how place-located identity influences people’s perceptions and behaviors toward that place, and studied its related constructs as regards to specifically located places (Giuliani and Scopelliti, 2009). For instance, “local identity” was applied to stress its location-related nature or in comparing to more global or abstract levels of place identity; “neighborhood identity” and “city identity” were used to address specifically place located identity concerning dwelling place at various scales (i.e., neighborhood and the city); “sense of community,” or probably the most general one “sense of place,” and “place attachment” focused on the affective relation of the individuals with their physical environment; and, “place dependence” focused on a functional dependence of an individual on specific places (Jorgensen and Stedman, 2001).

Human beings are exposed to various daily activities through physical and environmental settings, and the role of activities in defining the human–environment relationship has been well addressed within the field of environmental psychology. For instance, Lee and Shen (2013) found that involvement in leisure activities (e.g., walking their dogs in urban parks) predicts place loyalty; Bonaiuto et al. (2004) revealed that different sets of activities residents routinely performed in different places (i.e., resident’s neighborhood, city center, and suburb) relate to the ways people assess their neighborhood environmental quality and their neighborhood attachment; Bonaiuto et al. (1999) indicated that some indicators measuring the quality and quantity of the activities that are associated with a specific place can be related to neighborhood attachment too. Kyle and colleagues ran a lot of studies and they argued that hiking activity predicts an individual’s attachment to a specific place where people are engaged in hiking (Kyle et al., 2003, 2004a,b,c). They also found that engaging in hiking activity predicts one’s place identity (Kyle et al., 2004a), which can thus be considered as a cognitive link between the self-concept and the place where the activities are carried out (Hernández et al., 2010). As places where the daily activities are performed are not the same for each individual, each place has different meaning and importance for each individual, thus in the present study it was left decided for participants to freely indicate the place of reference for their self-definition, therefore adopting a non-specific place approach. The question is thus if place identity is promoted by an activity *per se* (e.g., in the above-mentioned case, the hiking activities), or if it is rather promoted by specific activity’s features. A theoretically based hypothesis would thus predict flow—experienced by people while they are doing such activities—as a crucial factor under this respect (Wöran and Arnberger, 2012). However, no empirical evidence at all on the place identity–flow link has been provided yet, thus making the present work the first one aiming to empirically test the relation between a located flow experience and the corresponding place identity, on the basis of the eudaimonistic identity theory (EIT).

### Eudaimonistic Identity Theory

EIT (Waterman, 1984, 1990a, 1992, 1993a, 2004) proposes that an individual starts to recognize elements of his/her own true self—including goals, values, interests, talents, and abilities (Erikson, 1968, 1980)—through participation in self-defining activities (Waterman, 2004, 2005; Coatsworth et al., 2005, 2006; Schwartz and Waterman, 2006; Sharp et al., 2007). EIT is grounded in both Aristotle’s eudaimonia [Greek: ɛύδαιμονία (eudaimonía)] and Erikson’s theories of identity (Erikson, 1968). The word “eudaimonia” has been interpreted in different ways as “*well-being*” (Ross, 1949), “*flourishing*” (Cooper, 1999), and “*happiness*” (Waterman, 1993b), and it has been used with reference to aspects of human flourishing: i.e., an intrinsically good state or activity essential for good life (Fraser, 2014), seeking to use and develop the best in oneself (Huta, 2012), the various realization of one’s potential (Irwin, 1985), the highest human good that arises from activities achievable by human action (Ryff and Singer, 2008), and to recognize and live in accordance with one’s “true self” (Norton, 1976). According to Aristotle, eudaimonia actually requires activity, action, so that it is not sufficient for a person to possess an unpotentiated ability or disposition. Aristotle’s teachings on eudaimonia have influenced psychological research regarding personal growth and fulfillment (i.e., Csikszentmihalyi, 1975, 1990; Deci and Ryan, 1985, 1987, 1991, 2002; Waterman, 1990a,b, 1992, 1993a, 2004; Diener and Lucas, 2000; Ryff and Singer, 2006; Huta, 2012). Whereas Erikson’s (1968) identity is regarded as goals, values, and beliefs central to a person’s self-definition. Thus, based on the philosophical underpinnings of eudaimonia and Erikson’s identity, EIT focuses on self-defining activities that are likely to influence identity, via the discovery and recognition of one’s domain or true self and by means of flourishing through one’s own interests, talents, and abilities. In short, EIT is identity discovery through participation in self-defining activities.

### Self-defining Activities

Self-defining activities are a category of activities that are representative of who an individual is as a person (Coatsworth et al., 2005). Waterman (2004) maintained that it is through the self-defining activities exploration that an individual forms the basis for building a coherent personal identity. Such self-defining activities—identified by an individual as being vital to who one is and what one is like as a person—are essential for identity consolidation. This happens especially during adolescence and emerging adulthood, for example, by providing a broad social identity (Eccles and Barber, 1999) and as they suggest appropriate behaviors and how one fits into the social milieu (Coatsworth et al., 2005; Sharp et al., 2007). Self-defining activities provide opportunities to explore whether a specific social identity is comfortable and consonant with one’s true self, and to explore whether it facilitates the necessary identity work for a true identity integration (Barber et al., 2001). A number of studies have provided evidence that self-defining activities may assist adults in exploring their sense of meaningful and coherent identity, by facilitating the necessary identity work useful for identity integration, and in providing a unique context for exploring interests and talents (Fredricks et al., 2002; Hansen et al., 2003; Sharp et al., 2007). Such evidence has in many senses supported Maslow’s (1970) statement that activities that are important for identity development are those that provide the impetus toward self-actualization or a full use and exploration of talents, capacities, and potentials. Such evidence has also expanded the sense in which self-defining activities are associated with identity in the context of activity, and it is further informative for understanding the features of an activity experience in connection with flow. However, empirical evidence on identity and flow is notably sparse, and the more specific relation between one’s own located flow experience and her/his own place identity cannot be traced in existing research records.

### State of the Literature

Theoretical underpinnings (i.e., flow, place identity, EIT, self-defining activities) introduced above provide the bases for a connection between activities and flow on the one side, and between activities and place identity on the other side. On such bases, it is theoretically plausible and meaningful to propose a link between experiencing flow in a place located activity and experiencing the corresponding place identity, that is, the psychological experience of the place where the activities are performed. However, to our knowledge, the empirical evidence on the association between flow experience and place identity is absent. In fact, the experience of the activities could induce not only flow, but also a feeling of personal expressiveness (Waterman, 1993b), when the activity provides an intense involvement; a feeling of special meshing with the activity; a feeling of being alive; complete or fulfilled; and a feeling of who one really is (Waterman, 1990a). Such feelings have been interpreted as the equivalent of Aristotle’s concept of eudaimonia (Waterman, 1990a,b, 1992), which calls on individuals to recognize and to realize their true potential (Norton, 1976; Aristotle, 1985). Moreover, the activities a person carries out are important for her/his own identity definition or strength (Coatsworth et al., 2005, 2006). Based on the EIT, activity experiences such as flow and personal expressiveness are conceptually linked (Waterman, 1993b) and their relations are already empirically addressed via quantitative correlational studies (Waterman et al., 2003; Coatsworth et al., 2005; Waterman, 2005; Schwartz and Waterman, 2006; Sharp et al., 2007). On these bases, personal identity, as a sub-construct of personal expressiveness (Waterman, 2005), and its relation with flow already received empirical demonstration (Mao et al., 2016a). Furthermore, a similar correlation had been proved between one’s own perceived social identity strength and flow when engaging in socially group activities (Mao et al., 2016b). Thus the present contribution attempts to provide the first quantitative evidence of such a link between a place-located flow experience and one’s own identification with his/her own favored place.

## Research Aims and Hypotheses

The present paper—based on the EIT in which identity discovery is generated from self-defining activities (e.g., Waterman, 1984, 1990a, 1992, 1993a, 2004), as well as on the flow theory (Csikszentmihalyi, 1975) in which flow is experienced in various daily activities by individuals in different walks of life—aims to extend to the place identity level what—based on previous investigations (Mao et al., 2016a,b)—had been already demonstrated regarding the link among flow and an individual’s identity at both the personal and the social level. Followed by Sharp et al. (2007) who claimed that multi-national design is crucial for understanding the generalization of models of identity development, we carried out our study in two nations in Europe, where we explored the association between an individual’s place located flow experience and his/her own perceived place identity related to that place. Those two samples had been selected on a convenient basis, in terms of availability within the existing research funding financed to one of the corresponding authors. This had been checked with reference to four self-defining activities that were engaged in one’s own specified place. Specifically, four self-defining activities (i.e., *Activity 1. Activity 2. Activity 3. Activity 4*) are derived—according to flow theory—from four combinations of an individual’s perceived skills (*low vs. high*) and related challenges (*low vs. high*). To realize this aim, the present work was guided by the following hypotheses:

H1: It is assumed that an individual’s perceived flow or place identity, would neither be affected by gender, nor by age, when engaging in one’s own reported self-defining activities from his/her own specified places where those activities are conducted.
H2: Based on the above reasoning, it is supposed that there is a main effect due to the different place specific self-defining activities (i.e., *Activity 1. Activity 2. Activity 3. Activity 4*) on both place located flow experience and place identity.
H3: Main Hypothesis: It is expected that participant’s perceived flow is positively associated with his/her own perceived place identity when engaging in place-specific self-defining activities.

## Materials and Methods

### Materials

Standard and specific instruments were used to measure an individual’s degree of flow experience and that of place identity, in sequence for four different place-specific self-defining activities, which are elicited by combinations of different levels of skills (*low vs. high*) and challenges (*low vs. high*). The instruments were delivered by means of a self-report questionnaire consisting of three sections. The first section included two open-ended questions for the generation of four self-defining activities with regard to the specific place where an individual would conduct such activities. The second section, which comprised 32 multiple-choice questions regarding flow and place identity, was administered separately for each of the above four different activities. The majority of the questionnaire items that were positively worded meant to express an individual’s perceived flow and place identity strength, while the other four negatively worded items were coded reversely. Responses to all of the multiple-choice questions were registered on a 5-point Likert-type scale with answers ranging from “*Not at all characteristic of me*” to “*completely characteristic of me.*” Finally, the third section consisted of 10 questions concerning participant’s social-demographic features.

### Self-defining Activities Instrument

**FIGURE 1:**
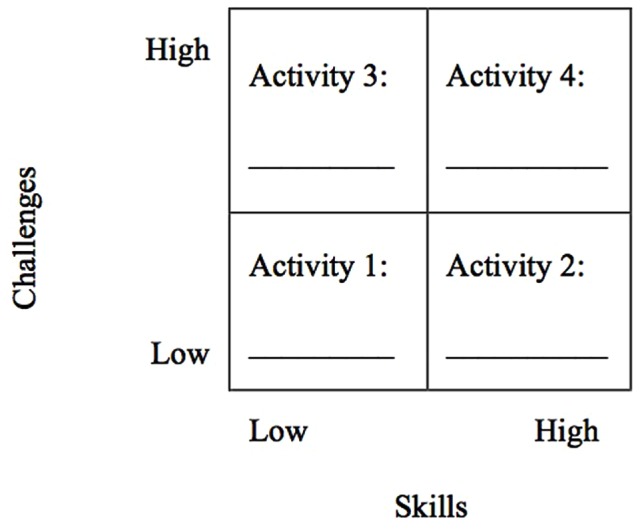
**The 2 (*skills vs. challenges*) by 2 (*low vs. high*) table for generating four activities**.

The standard version of the Personally Expressive Activities Questionnaire (PEAQ; Waterman, 1993b; Schwartz and Waterman, 2006) was used, which originally asked to identify five activities of importance to the respondent, that is, that he/she would use in describing himself/herself as who he/she is to another person. The instructions were modified in order for the participants to make an activity choice that pertained to the specific place. The instructions were thus as follows: *(a) If you wanted to make it clear to another person WHO YOU ARE and what you are like as a person BELONGING TO A PLACE (any: geographical, occupational, recreational, home, etc.), what activities which are important to you and in which you regularly engage in THAT PLACE would you describe (minimum 5 activities maximum 10 activities)?* The subsequent question required to select four activities from those 5 to 10 activities, and place them in a 2 (*skills vs. challenges*) × 2 (*low vs. high*) table (see **Figure 1**) by the relative standing, in the following order to fit the criteria for the channels: *Activity 1* (low skills low challenges—corresponding to *Apathy* in Experience Fluctuation Model, EFM; Csikszentmihalyi and Massimini, 1985; Massimini et al., 1988); *Activity 2* (high skills low challenges—corresponding to *Relaxation* in EFM); *Activity 3* (low skills high challenges—corresponding to *Anxiety*); *Activity 4* (high skills high challenges—corresponding to *Flow*). Specifically, the instructions read as below: *(b) From the above activities, please choose 4 (four) activities in which you regularly engage that are characterized by different combinations of skills (low vs. high) and challenges (low vs. high), and write the chosen activities in each of the following boxes*. Each activity (i.e., *Activity 1. Activity 2. Activity 3. Activity 4*) was then rated on the measures of flow and place identity.

### Flow Scale

An 8-item flow scale (item 1–8) that has been widely used by Waterman and colleagues (Waterman et al., 2003, 2008; Schwartz, 2006; Schwartz and Waterman, 2006) was applied to the present study. The items (e.g., “*I feel I have clear goals*,” “*I lose track of time*”) are corresponding to flow elements identified by Csikszentmihalyi (1990). Cronbach’s alpha coefficient based on the present sample is 0.90 for Greek participants, and 0.83 for Italian respondents.

### Place Identity Tools

Given the multifaceted nature of the place identity construct and its several available relevant scales, multiple tools were used, then their internal consistency of Cronbach’s alpha and interelations had been tested in order to identify which tool to adopt in the subsequent analyses aiming at testing the present contribution’s hypotheses.

#### Sense of Place (12 Items: Item 9–20)

The 12-item “sense of place” scale is composed of three sub-scales: “place identity” (four items: 9–12; i.e., *I feel that I can be myself at my lake property*), “place attachment” (four items: 13–16; i.e., *I feel relaxed when I’m at my lake property*), and “place dependence” (four items: 17–20; i.e., *My lake property is the best place for doing the things that I enjoy most*). The scale was developed by Jorgensen and Stedman (2001) based on previous existing research (Williams and Roggenbuck, 1989; Stedman, 1997). It is adapted in the current study by replacing “lake property” with “this place,” so that in the present work the items pertained to the perception of each individual’s own specifically reported places (e.g., “*I feel relaxed when I am at this place*,” “*I feel that I can really be myself at this place*”). Cronbach’s alpha for each subscale was 0.76, 0.84, and 0.74, respectively (Jorgensen and Stedman, 2001), while for the present sample, it is respectively 0.56 (or 0.84 if item 10 is deleted), 0.87, and 0.58 for Greek participants, and 0.71 (or 0.85 if item 10 is deleted), 0.86, and 0.74 for Italian participants.

#### Neighborhood Attachment (Four Items: 21–24)

The Fornara et al.’s (2010) 4-item neighborhood attachment scale was modified, so that items pertained to the participants’ sense of attachment to their own specific place (e.g., “*it would be very hard to leave from this place*,” “*I do not feel integrated in this place*”). Participants responded to four items designed to assess their feelings, bonds, thoughts, and behavioral intentions with reference to their social–physical environment. Cronbach’s alpha coefficient was reported as 0.82 (Fornara et al., 2010) in a previous study, however, in the present work, the internal consistency is respectively 0.61 (or 0.79 if item 24 deleted) and 0.71 (or 0.86 if item 24 deleted) for the Greek and Italian sample.

#### Local Identity (Single Item 25)

Adapted from Bonaiuto et al.’s (1996) research on local identity, the question: “*Do you feel proud of living in this town* (7-point scale: ‘*not at all*’ to ‘*very much*’)” was modified into a statement “*I feel proud of living in this place*,” so that it pertained to the current work referring different people’s specified favored place. Responses were registered on a 5-point Likert-type scale ranging from “*not at all characteristic of me*” to “*completely characteristic of me*.”

#### Sense of Community (Seven items: 26–32)

The selected 7-item “sense of community” scale was developed by Prezza et al. (2001). Following the research on Prezza et al.’s (1999) *Italian Scale of Sense of Community* (which is translated and modified from *Sense of Community Scale* by Davidson and Cotter, 1986)—was modified in order to fit the current research context. The Italian Scale of Sense of Community includes 18 items (Cronbach’s alpha = 0.82); however, in the current study only seven of these items were adapted (e.g., “*I feel safe in this place*,” “*When I travel, I’m proud to tell others this place*”). The internal consistency reliability estimate for scores is 0.90 for the Greek sample, and 0.92 for the Italian sample.

Statistical evidence based on the present Greek and Italian sampling data, shows that within the “sense of place” scale, its sub-scale of “place identity” (three items: 9—*Everything about this place is a reflection of me, 11—I feel that I can really be myself at this place, 12—This place reflects the type of person I am*) correlated well with some other sub-scales like “place attachment” (*r*_Gr_ = 0.82; *r*_It_ = 0.72), and “place dependence” (*r*_Gr_ = 0.40; *r*_It_ = 0.58). Furthermore this “place identity” sub-scale also correlated with the other literature derived scales such as “neighborhood attachment” (*r*_Gr_ = 0.56; *r*_It_ = 0.57), “local identity” (*r*_Gr_ = 0.47; *r*_It_ = 0.65), and “sense of community” (*r*_Gr_ = 0.56; *r*_It_ = 0.67). Most importantly, the “place identity” scale is more in line with the place identity construct to which the present research aims. Consistently with the previous two studies which tested the link of personal and social identity to flow experiences (Mao et al., 2016a,b), the selected 3-item “place identity” sub-scale among the “sense of place” measures, therefore, is chosen for the further analyses testing the main hypothesis about the flow–place identity association.

### Methods

#### Participants

A total of 287 convenient sample—134 local residents from Thessaloniki (the second largest city in Greece) and 153 residents from Rome (the capital city of Italy)—participated in present study. Participant’s socio-demographic features were reported in **Table 1.** Informed consent was obtained from each subject after the explanation of the study, which was approved by the local institutional ethical committee (Institutional Review Board of *Sapienza University of Rome* and *Alexander Technological Educational Institute of Thessaloniki*).

**Table 1:** Socio-demographic features of the sample.

Country	*N*	Gender	Age		Educational degree				Region	
					
		Male (%)	*M_age_* (age range)	SD	High school (%)	Bachelor (%)	Master (%)	Ph.D. (%)	Urban (%)	Sub-urban (%)
Gr	134	50.7	28.5 (18–58)	9	38.8	25.4	17.9	17.9	74.6	25.4
It	153	51.6	31 (19–59)	9	34.6	22.9	36.6	5.9	72.5	27.5


#### Procedures

Firstly, the original English-language questionnaire was submitted to the standard translation and back-translation procedure (Brislin, 1970)—i.e., it was translated from English into Greek and Italian—and subsequently the Greek and Italian version questionnaires were respectively translated back into English. Then the comparison among the original and the new English translation was made, and a few necessary modifications were conducted; finally, the synthesized version was launched online and piloted for about 10 subjects. The Greek questionnaire was administered via Qualtrics Survey Software, while the survey link of the Italian questionnaire was created in the Google Docs environment. These two hyperlinks were sent to the potential respondents via Email, Facebook, and Skype by the research group. Data collection started once each participant’s informed consent was provided: data gathering happened from March to June 2014. Statistical analyses were performed via SPSS Statistics 20.0.

### Data Analytic Strategies

#### Preliminary Analysis

To understand if gender or age, as independent factors, could affect an individual’s subjective experience of flow and his/her perceived place identity strength, an *Independent Sample T-Test* was applied to test gender differences on flow and place identity, while *Univariate Analyses of Variance* were conducted to test age differences separately on both flow and place identity.

#### Manipulation Check on Flow

In accordance with the 2 by 2 research design in which four different self-defining activity types were generated on the basis of the different combinations of skills (*low vs. high*) and challenges (*low vs. high*), a main effect of activity type (four differentially self-defining activity types) on the individual’s perceived flow is anticipated. Specifically, flow experience was compared across different activity types (i.e., *Activity 1. Activity 2. Activity 3. Activity 4*) with four-level (four activity types) *one-way repeated measures of analysis of variance* (*ANOVA*). When the main effect was significant (*p* < 0.05), the *post hoc* pairwise comparison via Bonferroni approach would be applied to test the varying trend of flow across these four different levels of the activity.

#### Pearson Correlations between Flow and Place Identity

To test the main hypothesis, for each activity (*Activity 1*—*Apathy. Activity 2*—*Relaxation. Activity 3*—*Anxiety. Activity 4*—*Flow*) the associations among flow and place identity were assessed by calculating their *Bravais–Pearson Correlation Coefficients* with a two-tailed test of significance using the method of bivariate correlation. Afterward, the same method was applied to test the overall correlation between both constructs across all activity types based on the individual level.

## Results

### Preliminary Results

#### Gender Effect on Flow and Place Identity

**Table 2** presents Only in the premise that there were not statistically significant differences observed in the preliminary analyses, were gender, and age then combined for subsequent manipulation check and correlational tests. mean values and standard deviations that were disaggregated by gender: results yielded from the *Independent Sample T-Test* for gender difference indicated no significant difference on flow (all *p*s > 0.05), yet no significant difference on place identity (all *p*s > 0.05). Thus gender was neither related with flow nor with place identity, as hypothesized in H1.

**Table 2:** Gender differences in flow and place identity.

	Sample by country	Males	Females	*t*	*p*	Effect size *d*
Flow	Greek	3.32 (0.62)	3.45 (0.65)	-1.16	0.25 (n.s.)	0.001
	Italian	3.56 (0.46)	3.75 (0.56)	-2.32	0.08 (n.s.)	0.035
Place identity	Greek	3.46 (0.71)	3.50 (0.75)	-3.78	0.71 (n.s.)	0.001
	Italian	3.37 (0.68)	3.61 (0.85)	-2.04	0.10 (n.s.)	0.027


#### Age Effect on Flow and Place Identity

**Table 3** shows mean values and standard deviation disaggregated by age classes. *Univariate Analyses of Variance* with age as independent variable did not show any significant effects (all *p*s > 0.05) on flow or place identity concerning the Greek sample (recoded as follows: 18–29; 30–39; 40–49; 50–58), nor concerning the Italian sample (i.e., 19–29; 30–39; 40–49, 50–59). Thus, again in line with H1, even age was unrelated to flow and place identity, as hypothesized.

**Table 3:** Age differences on flow and place identity regarding the two different samples.

Dependent variable	Sample by country	18–29	30–39	40–49	50–59	*F*	*p*	Partial eta squared ($\eta_{p}^{2}$)
Flow	Greek	3.42 (0.68)	3.26 (0.52)	3.38 (0.48)	3.50 (0.67)	0.57	0.63	0.013
	Italian	3.61 (0.55)	3.80 (0.41)	3.49 (0.53)	3.60 (0.61)	1.89	0.14	0.037
Place identity	Greek	3.51 (0.75)	3.30 (0.66)	3.68 (0.73)	3.75 (0.94)	1.08	0.36	0.024
	Italian	3.41 (0.77)	3.71 (0.76)	3.36 (0.83)	3.47 (0.74)	1.58	0.20	0.031


#### Results for Manipulation Check on Flow

**Table 4** presents the effect of activity type (four types: *Activity 1. Activity 2. Activity 3. Activity 4*) on the participant’s perceived flow experience: one-way repeated ANOVA, with activity as the design factor, revealed significant difference on flow for both of the Greek participants [*F*(3,134) = 41.30, *p* < 0.001], and the Italian respondents [*F*(3,153) = 13.91, *p* < 0.001]. Specifically, for both sample, the mean value for flow experience on each activity indicated a growing trend from *Activity 1* to *Activity 4*. The mean value for flow within both samples manifested that flow experience is best achieved when both skills and challenges are at the peak level, i.e., when engaging in *Activity 4*. Importantly, “high challenge-high skill” activity (*Activity 4*) was associated with greater reported level of flow than “high challenge-low skill” activity (*Activity 3*).

**Table 4:** The descriptive statistics of scores on flow scale.

Activity type		*N*	Mean	Standard deviation	Standard error	Minimum	Maximum	*F*	*p*
Greek sample	1.00	134	2.81	1.07	0.09	1.00	5.00	41.30	0.000
	2.00	134	3.23	0.86	0.07	1.25	5.00		
	3.00	134	3.50	0.70	0.06	1.13	5.00		
	4.00	134	3.98	0.88	0.08	1.25	5.00		
	Total	536	3.38	0.98	0.04	1.00	5.00		

Italian sample	1.00	153	3.39	0.70	0.06	1.13	1.13	13.91	0.000
	2.00	153	3.63	0.77	0.06	1.00	1.00		
	3.00	153	3.65	0.72	0.06	1.00	1.00		
	4.00	153	3.93	0.76	0.06	1.63	1.63		
	Total	612	3.65	0.76	0.03	1.00	1.00		


*Post hoc* analyses using the Bonferroni criterion for significance (see **Table 5**) manifested that the pairwise differences on flow among activities for the Greek sample were: Flow_1_ < Flow_2_ (*p* < 0.001), Flow_1_ < Flow_3_ (*p* < 0.001), Flow_1_ < Flow_4_ (*p* < 0.001), Flow_2_ < Flow_3_ (*p* < 0.005), Flow_2_ < Flow_4_ (*p* < 0.001), Flow_3_ < Flow_4_ (*p* < 0.001). For the Italian sample, the similar trend of differences was obtained: Flow_1_ < Flow_2_ (*p* < 0.005), Flow_1_ < Flow_3_ (*p* < 0.005), Flow_1_ < Flow_4_ (*p* < 0.001), Flow_2_ < Flow_3_ (*p* = 0.749), Flow_2_ < Flow_4_ (*p* < 0.001), Flow_3_ < Flow_4_ (*p* = 0.001). The varying trend of the flow experience across the four activities from the two samples followed this pattern: *Flow* > *Anxiety* > *Relaxation* > *Apathy*.

**Table 5:** Multiple comparisons of flow among four activity types.

Dependent variable	(*I*) Activities	(*J*) Activities	Mean difference (*I*–*J*)	Standard error	*p*
Greek (*N* = 134)	1 (*Apathy*)	2	-0.42^∗^	0.11	0.000
		3	-0.69^∗^	0.11	0.000
		4	-1.17^∗^	0.11	0.000
	2 (*Relaxation*)	1	0.42^∗^	0.11	0.000
		3	-0.27^∗^	0.11	0.014
		4	-0.75^∗^	0.11	0.000
	3 (*Anxiety*)	1	0.69^∗^	0.11	0.000
		2	0.27^∗^	0.11	0.014
		4	-0.49^∗^	0.11	0.000
	4 (*Flow*)	1	1.179^∗^	0.11	0.000
		2	0.75^∗^	0.11	0.000
		3	0.49^∗^	0.11	0.000

Italian (*N* = 153)	1 (*Apathy*)	2	-0.23^∗^	0.08	0.006
		3	-0.26^∗^	0.08	0.002
		4	-0.54^∗^	0.08	0.000
	2 (*Relaxation*)	1	0.23^∗^	0.08	0.006
		3	-0.03	0.08	0.749
		4	-0.31^∗^	0.08	0.000
	3 (*Anxiety*)	1	0.26^∗^	0.08	0.002
		2	0.03	0.08	0.749
		4	-0.28^∗^	0.08	0.001
	4 (*Flow*)	1	0.54^∗^	0.08	0.000
		2	0.31^∗^	0.08	0.000
		3	0.28^∗^	0.08	0.001


*^∗^The mean difference is significant at the 0.05 levels 1, 2, 3, and 4 are *Apathy*, *Relaxation. Anxiety*, and *Flow*, respectively.*

On the whole, these results support the case that the four activity types are relatively different in terms of the perceived degrees of flow. On the basis of the previous study by Waterman et al. (2003), it is not difficult to understand that the varying degree of flow is related correspondingly to varying degrees of both skills and challenges. Moreover, the manipulation check has proved that the instruments for flow are an effective measure to distinguish four different activity types by relative standing based on combinations of different levels of skills by challenges. And such a manipulation check has proved that the activity type indeed has different characters (i.e., *Apathy. Relaxation. Anxiety. Flow*).

#### Pearson Correlations between Flow and Place Identity

**Table 6** presents the correlation coefficients between flow and place identity, both on the activity level (*Activity 1* to *Activity 4*) and on the individual level (considering all activity types together). Detailed findings are reported via each activity type. In the *Apathy*—the low skills and low challenges combination—the correlations between the individual’s flow experiences and place identity were significant and positive as expected, specifically, concerning the Greek sample (*r* = 0.80, *p* < 0.001) and for the Italian sample (*r* = 0.62, *p* < 0.001). In the *Relaxation*—the high skills and low challenges combination—the correlations between the individual’s flow experience and place identity were significant and positive; specifically, for the Greek sample (*r* = 0.69, *p* < 0.001), and for the Italian sample (*r* = 0.56, *p* < 0.001). In the *Anxiety*—the low skills and high challenges combination—the correlations between the individual’s flow experiences and place identity were significant and positive, also, *r* = 0.49, *p* < 0.001 for the Greek sample, and *r* = 0.44, *p* < 0.001 for the Italian sample. In the *Flow*—the high skills and high challenges combination—the correlation of the individual’s flow experiences to place identity were significant and positive for the Greek sample (*r* = 0.65, *p* < 0.001), and for the Italian sample (*r* = 0.50, *p* < 0.001).

**Table 6:** Correlations between flow and place identity on both of the activity level and individual level.

	Correlation	Greek sample (*N* = 134)	Italian sample (*N* = 153)
Activity 1 (*Apathy*)	Flow	0.80^∗∗^	0.62^∗∗^
	Place identity		
Activity 2 (*Relaxation*)	Flow	0.69^∗∗^	0.56^∗∗^
	Place identity		
Activity 3 (*Anxiety*)	Flow	0.49^∗∗^	0.44^∗∗^
	Place identity		
Activity 4 (*Flow*)	Flow	0.65^∗∗^	0.50^∗∗^
	Place identity		
Across activities	Flow	0.61^∗∗^	0.55^∗∗^
	Place identity		


*^∗∗^Correlation is significant at the 0.01 level (two-tailed).*

Due to the fact that flow is the subjective experience based on the individual’s personal perception, and perceived place identity *per se* is a personal level variable, an additional correlation on the individual level was calculated and indicated in **Table 3.** Similar to the previous four correlational situations, the correlation between perceived flow and perceived place identity was significant (*p* < 0.001). Specifically, the correlation in the Greek sample was 0.61, while for the Italian sample was 0.55.

## Discussion

The present work draws upon research in EIT, flow theory, and place identity theory, to organize a unifying framework for how the individuals’ optimal experience is likely to be associated with place identity (their identification of who they personally are in terms of their belongingness to a place they prefer), by means of engaging in self-defining activities. Among all of those place-located self-defining activities reported on the basis of four different combinations of skills (*low vs. high*) by challenges (*low vs. high*), and via two different samples, some work, or study related place self-defining activities were listed (i.e., programming in office at university campus, reading or writing at the corner of a coffee bar). There are some other types of place-located activities too: for instance, to regularly participate a choir in a nearby church, weekend dinner with family and friends at seaside restaurants, playing football at Olympic Sports Playground in Thessaloniki, attending opera performance at *Teatro dell’Opera di Roma*, watching the World Cup at the city center and drinking beers with friends, attending charity symphony to perform/donate for Syrian refuge at *Aristotle University*, and so on and so forth. Those activities performed in various places helped define an individual’s identity in relation to the physical environment by means of a complex conscious or unconscious feelings, beliefs, ideas, interests, goals, values, and skills relevant to the environment (Proshansky, 1978), while indicated that places in which all of these activities performed, helped adding environmental meanings to one’s self-identity via his/her preferably selected places as who they are (Csikszentmihalyi and Rochberg-Halton, 1981).

To our knowledge, this is the first study to connect the flow construct from positive psychology, with place identity from environmental psychology. Results obtained from the present study enhance the existing literature, by suggesting that being involved in place-located activities that bring people to flow is related to their personal growth in a specific place. The relation of the activity experience (like flow) with respect to the sense of identity, the gist of EIT, has been usually focused on the personal identity level (Waterman, 1990a, 1992, 1993a; Waterman et al., 2003; Schwartz, 2006; Schwartz and Waterman, 2006; Mao et al., 2016a), and sparsely on the social identity level (Mao et al., 2016b). However, the present contribution has extended this to the place level: the findings support our main hypothesis and are in line with the previous limited studies.

First, results from the preliminary analyses indicate that the effects of either gender or age have no impact on the individual’s subjective experience of flow. Such results confirm the previous descriptive findings from various countries and cultures by Csikszentmihalyi (1990), who suggested that flow experience is universal and cuts across gender and age (Csikszentmihalyi, 1975/2000). It has also reaffirmed some other empirical research findings that are generated from small college student samples, according to which gender did not play role in the subjective experience of flow (Russell, 2001; Martin and Cutler, 2002; Delle Fave and Massimini, 2005). Moreover, the individual’s subjective identity experience (perceived place identity strength induced from self-defining activities) was consistently found irrespective of age or gender. Such a finding has enriched the EIT (Waterman, 1990a,b, 1992, 1993a, 2005, 2007; Waterman et al., 2008), by expanding the activity–personal identity relationship to the place identity level.

Secondly, as regards to the manipulation check on flow, the combinations of skills by challenges created four different activity types (*Activity 1. Activity 2. Activity 3. Activity 4*) corresponding to EFM (*Apathy. Relaxation. Anxiety. Flow*): they resulted in significantly different levels of reported flow among four different activity types (*p* < 0.001). Specifically, the subjective flow experience observed both from the Greek and Italian sample, confirmed the hypotheses and it had been increasingly produced moving from *Activity 1* (*Apathy*) to *Activity 2* (*Relaxation*), to *Activity 3* (*Anxiety*), up to *Activity 4* (*Flow*): it theoretically confirmed that flow, involving both skills and challenges, is best achieved when skills and challenges are balanced and are above average level (Csikszentmihalyi and Csikszentmihalyi, 1988; Csikszentmihalyi and LeFevre, 1989; Csikszentmihalyi, 1990). In this sense, flow experience can be considered to be associated with eudaimonia, as flow is conceived to be inherently growth-producing by making use of one’s skills and abilities, while also leading to their progressive development (Nakamura and Csikszentmihalyi, 2002, 2009; Waterman, 2007). This manipulation check lent credence to both the activity classification system as well as the convergence of these categories with reported experiences of individual’s flow, which was assessed by means of the flow measure characterized by some key components of flow proposed by Csikszentmihalyi (1975, 1990). The self-defining activities which participants freely listed, and selected to place in the 2 by 2 table, being characterized by the combinations of different levels of skills and challenges, are more or less associated with flow experience, and are most prominent in *Activity 4*. This is in keeping with the foundations of the flow literature (Csikszentmihalyi, 1975, 1990; Csikszentmihalyi and Csikszentmihalyi, 1988); furthermore, as increasingly challenging activities are enjoyed, the individual is more likely to develop an identity that is experienced with increased complexity and control (Csikszentmihalyi and Rochberg-Halton, 1981) when in the state of flow.

With regard to the main hypothesis on flow–place identity relationship, the significant and positive correlations revealed from Bravais–Pearson bivariate correlation between flow and place identity have been confirmed within two different samples from two different countries. Such a finding is found to hold across four different activity types and it strongly supports that flow, as an optimal experience, holds across lines of countries, age, gender, and activity types (Nakamura and Csikszentmihalyi, 2002; Mao et al., 2016a). Thus, place-located activities producing increased perceptions of flow are associated with increased reports of place-specified identity. Statistically speaking, comparing the data gathered from these two countries, the highest correlation between place-located flow and place identity is found in Greece; this result has supported previous findings conducted in the same city of Thessaloniki: notwithstanding the highest financial crisis in Greece, its residents’ health status, work-life balance, and personal security are above average (OECD, 2015), and they still conceive their city as an ideal place as it meets the standards of living, provides good quality of life, offers varieties of choices in terms of entertainment and leisure activities, and, more importantly, they did not wish to leave their city (Dimitrakopoulou et al., 2013). This confirmation of the primary hypothesis provides support for extending the implications of EIT (Waterman, 1990a, 1992, 1993a, 2004, 2005) into the social domain at the residential or other environmental settings where people engage in flow activities for enjoyment. In this regard, flow experience can be conceived as eudaimonic, since it can be intrinsically enjoyable (Waterman, 1993a; Seligman, 2002), and due to its role in increasing intrinsic motivation, where subjective experiences of flow are thought to organize and activate thoughts and behaviors that contribute to the affirmation and construction of self-definitions. The relationship between one’s own place-located flow and the enhanced bonds with one’s own place shows that individuals’ positive self-esteem and emerging identity can be coupled with a place and its related functions, and the identifications with a place can be seen as similar to social identification with a group, according to identity principles and coping strategies (Bonaiuto et al., 1996; Dixon and Durrheim, 2000). Moreover, such correlations may support that the physical properties of places impose behavioral requirements (e.g., conducting enjoyment activities) on the individual that may in turn have important effects on the formation or development of place identity (Proshansky, 1978; Proshansky et al., 1983; Proshansky and Fabian, 1987). This is in accordance with previous literature on place identity purporting that it is induced from specific activities (e.g., leisure activities; Kyle et al., 2004a). Also, such correlations have implications for developing the constructs of place identity and place attachment (Bonnes et al., 2003; Giuliani, 2003), thus situating this work on the flow–place identity link with prior positive psychology work in contributing to the environmental psychology.

Finally, this study has limitations that warrant notice. First, the samples, expanded from the previous limited range of ages (Mao et al., 2016a), span differing demographic backgrounds, representative of distinct cities from two countries, as made up of a wide range of occupations. These features may provide regional confounds while also limiting the representativeness of the findings. Second, given the correlational nature of the present study design, it remains difficult to locate the causal inference, i.e., does a person’s perceived place identity strength facilitates the embedded flow experience, or is it the case that experiencing flow in an urban/suburban setting, results in stronger place identity strength? The possibility remains in the former case (Mao et al., 2016a,b) that those self-defining activities in which participants have developed a sense of place identity may provide the proper context for flow experiences. Well-developed relationships with others, for instance, may provide “safe harbors” for flow experience (Delle Fave et al., 2011). These interpretations are not mutually exclusive, however, and the potential also remains for place-located, multidirectional influences such that place identity both fosters and develops through flow experiences. At present, current literature lacks experimental studies that might assert directionality of the observed effects.

Hence, future research may profitably explore the causal relationships between flow and place identity. Place-located flow and place identity interrelation as a potential mutually supportive link may contribute to the extension of the EIT beyond the personal level. As findings revealed in this study, flow experience may not only produce individual complexity, but also contribute to cultural complexity. Thus, flow may provide a pathway for the development of both individuation and integration in a manner that fosters place bonds in a positive way by contributing to a balance of healthy individual agency and development in combination with well-being in the physical environment. Continued investigations also allow for pinning down the associations between place-located flow and place identity at specific levels, i.e., place attachment, residential satisfaction, place loyalty (i.e., when dealing with leisure and touristic activities), or at a more social level, i.e., ethnicity and religion, nationality, political affiliation, vocations and avocations, stigmatized identities, relationships, as well as engagement within larger and non-exclusive community ties and cultural groups or with nature in general and thus with natural places and areas. When engaging in an activity that requires personal skills, and yet in which the experience is challenging, individuals may seek the opportunity to develop both themselves and the links with the larger environment to which they belong. In conclusion, the findings reported here demonstrate that when flow is experienced within a place via self-defining activities, the sense of identity at the place level is also experienced and is seen significantly stronger in comparison to those cases in which activities are less conducive to flow.

## Author Contributions

MB, YM, and MC were involved in the planning of the study; YM, AP, SA, and UG conducted the data collection; YM did the data analysis and the writing of the article. MB, YM, SR, AP, and MC did the manuscript revision.

## Conflict of Interest Statement

The authors declare that the research was conducted in the absence of any commercial or financial relationships that could be construed as a potential conflict of interest.

The reviewer MB declared a shared affiliation, though no other collaboration, with the authors MB and SA to the handling Editor, who ensured that the process nevertheless met the standards of a fair and objective review.

## References

[B1] Aristotle (1985). *Nicomachean Ethics. (T. Irwin, Trans.).* Indianapolis, IN: Hackett.

[B2] Barber B. L. Eccles J. S. Stone M. R. (2001). Whatever happened to the Jock, the brain, and the princess? Young adult pathways linked to adolescent activity involvement and social identity. *J. Adolesc. Res.* 16 429–455. 10.1177/0743558401165002

[B3] Bonaiuto M. Aiello A. Perugini M. Bonnes M. Ercolani A. P. (1999). Multidimensional perception of residential environment quality and neighbourhood attachment in the urban environment. *J. Environ. Psychol.* 19 331–352. 10.1006/jevp.1999.0138

[B4] Bonaiuto M. Bonnes M. (2000). “Social-psychological approaches in environment-behavior studies. Identity theories and the discursive approach,” in *Theoretical Perspectives in Environment-Behavior Research: Underlying Assumptions, Research Problems, and Methodologies*, eds Wapner S. Demick J. Yamamoto T. Minami H. (New York, NY: Kluwer Academic Plenum), 67–78.

[B5] Bonaiuto M. Bonnes M. Continisio M. (2004). Neighborhood evaluation within a multi-place perspective on urban activities. *Environ. Behav.* 36 41–69. 10.1177/0013916503251444

[B6] Bonaiuto M. Breakwell G. M. Cano I. (1996). Identity processes and environmental threat: the effects of nationalism and local identity upon perception of beach pollution. *J. Community Appl. Soc. Psychol.* 6 157–175. 10.1002/(SICI)1099-1298(199608)6:3<157::AID-CASP367>3.0.CO;2-W

[B7] Bonnes M. Bonaiuto M. (2002). “Environmental psychology: from spatial-physical environment to sustainable development,” in *Handbook of Environmental Psychology*, eds Bechtel R. B. Churchman A. (New York, NY: Wiley), 28–54.

[B8] Bonnes M. Lee T. Bonaiuto M. (2003). “Theory and practice in environmental psychology–An introduction,” in *Psychological Theories for Environmental Issues*, eds Bonnes M. Lee T. Bonaiuto M. (Aldershot: Ashgate), 1–25.

[B9] Brislin R. W. (1970). Back-translation for cross-cultural research. *J. Cross Cult. Psychol.* 1 185–216. 10.1177/135910457000100301

[B10] Coatsworth J. D. Palen L. A. Sharp E. H. Ferrer-Wreder L. A. (2006). Self-defining activities, expressive identity, and adolescent wellness. *Appl. Dev. Sci.* 10 157–170. 10.1207/s1532480xads1003_5

[B11] Coatsworth J. D. Sharp E. H. Palen L. Darling N. Cumsille P. Marta E. (2005). Exploring adolescent self- defining leisure activities and identity experiences across three countries. *Int. J. Behav. Dev.* 29 361–370. 10.1177/01650250500166972

[B12] Cooper J. M. (1999). “The psychology of justice in plato,” in *Reason and Emotion. Essays on Ancient Moral Psychology and Ethical Theory*, ed. Cooper J. M. (Princeton: Princeton University Press), 138–149.

[B13] Craig S. D. Graesser A. C. Sullins J. Gholson B. (2004). Affect and learning: an exploratory look into the role of affect in learning with autotutor. *J. Educ. Media* 29 241–250. 10.1080/1358165042000283101

[B14] Csikszentmihalyi M. (1975). *Beyond Boredom and Anxiety.* San Francisco, CA: Jossey-Bass.

[B15] Csikszentmihalyi M. (1982). “Towards a psychology of optimal experience,” in *Annual Review of Personality and Social Psychology* Vol. 3 ed. Wheeler L. (Beverly Hills, CA: Sage), 13–36.

[B16] Csikszentmihalyi M. (1990). *Flow: The Psychology of Optimal Experience.* New York, NY: Harper & Row.

[B17] Csikszentmihalyi M. (1997). *Finding Flow: The Psychology of Engagement with Everyday Life.* New York, NY: Basic Books.

[B18] Csikszentmihalyi M. Csikszentmihalyi I. (eds). (1988). *Optimal Experience.* Cambridge: Cambridge University Press.

[B19] Csikszentmihalyi M. Kleiber D. A. (1991). “Leisure and self-actualization,” in *Benefits of Leisure*, eds Driver O. L. Brown P. J. Peterson G. L. (State College, PA: Venture Publishing), 91–102.

[B20] Csikszentmihalyi M. LeFevre J. (1989). Optimal experience in work and leisure. *J. Pers. Soc. Psychol.* 56 815–822. 10.1037/0022-3514.56.5.815 2724069

[B21] Csikszentmihalyi M. Massimini F. (1985). On the psychological selection of bio-cultural information. *New Ideas Psychol.* 3 115–138. 10.1016/0732-118X(85)90002-9

[B22] Csikszentmihalyi M. Rochberg-Halton E. (1981). *The Meaning of Things.* London: Cambridge University Press.

[B23] Csikszentmihalyi M. (1975/2000). *Beyond Boredom and Anxiety.* San Francisco: Jossey-Bass.

[B24] Davidson W. B. Cotter P. R. (1986). Measurement of sense of community within the sphere of city. *J. Appl. Soc. Psychol.* 16 608–619. 10.1111/j.1559-1816.1986.tb01162.x

[B25] Deci E. L. Ryan R. M. (1985). *Intrinsic Motivation and Self-Determination in Human Behavior.* New York, NY: Plenum Press.

[B26] Deci E. L. Ryan R. M. (1987). The support of autonomy and the control of behavior. *J. Pers. Soc. Psychol.* 53 1024–1037. 10.1037/0022-3514.53.6.1024 3320334

[B27] Deci E. L. Ryan R. M. (1991). “A motivational approach to self: integration in personality,” in *Nebraska Symposium on Motivation: Perspectives on Motivation* Vol. 38 ed. Dienstbier R. (Lincoln, NE: University of Nebraska Press), 237–288. 2130258

[B28] Deci E. L. Ryan R. M. (2002). *Handbook of Self-Determination Research.* Rochester, NY: University of Rochester Press.

[B29] Delle Fave A. Massimini F. (2005). The investigation of optimal experience and apathy: developmental and psychosocial implications. *Eur. Psychol.* 10 264–274. 10.1027/1016-9040.10.4.264

[B30] Delle Fave A. Massimini F. Bassi M. (2011). “Acculturation and optimal experience,” in *Psychological Selection and Optimal Experience Across Cultures: Social Empowerment Through Personal Growth*, eds Delle Fave A. Massimini F. Bassi M. (Dordrecht: Springer), 273–293.

[B31] Demerouti E. (2006). Job characteristics, flow, and performance: the moderating role of conscientiousness. *J. Occup. Health Psychol.* 11 266–280. 10.1037/1076-8998.11.3.266 16834474

[B32] Diener E. Lucas R. E. (2000). “Subjective emotional well-being,” in *Handbook of Emotions*, 2nd Edn, ed. Lewis M. (New York, NY: Guilford), 325–337.

[B33] Dimitrakopoulou V. Drougkelidis F. Koutsovasili I. Loukri D. Papachronopoulou D. Chronopoulou M. (2013). *Young People’s Views and Expectation of Local Development*. Available at: http://www.seedcenter.gr/conferences/Crisis2014/papers/Δημητρακοπούλου%20et%20al_αυτιλήψεις%20και%20προσδοκίες%20των%20νέων%20της%20θεσσαλονίκης%20για%20την%20τοπική%20ανάπτυξη.pdf

[B34] Dixon J. Durrheim K. (2000). Displacing place-identity: a discursive approach to locating self and other. *Br. J. Soc. Psychol.* 39 27–44. 10.1348/014466600164318 10774526

[B35] Eccles J. S. Barber B. L. (1999). Student council, volunteering, basketball, or marching band: what kind of extracurricular involvement matters? *J. Adolesc. Res.* 14 10–43. 10.1177/0743558499141003

[B36] Erikson E. H. (1968). *Identity: Youth and Crisis.* New York, NY: Norton.

[B37] Erikson E. H. (1980). *Identity and The Life Cycle.* New York, NY: Norton.

[B38] Fornara F. Bonaiuto M. Bonnes M. (2010). Cross-validation of abbreviated Perceived Residential Environment Quality (PREQ) and Neighborhood Attachment (NA) indicators. *Environ. Behav.* 42 171–196. 10.1177/0013916508330998

[B39] Fraser C. (2014). Wandering the way: a eudaimonistic approach to the Zhuāngziǐ. *Dao* 13 541–565. 10.1007/s11712-014-9402-1

[B40] Fredricks J. A. Alfeld-Liro C. J. Hruda L. Z. Eccles J. S. Patrick H. Ryan A. M. (2002). A qualitative exploration of adolescents’ commitments to athletics and the arts. *J. Adolesc. Res.* 17 68–97. 10.1177/0743558402171004

[B41] Giuliani M. V. (2003). “Theory of attachment and place attachment,” in *Psychological Theories for Environmental Issues*, eds Bonnes M. Lee T. Bonaiuto M. (Aldershot: Ashgate), 137–170.

[B42] Giuliani M. V. Scopelliti M. (2009). Empirical research in environmental psychology: past, present, and future. *J. Environ. Psychol.* 29 375–386. 10.1016/j.jenvp.2008.11.008

[B43] Hamari J. Shernoff D. J. Rowe E. Coller B. Asbell-Clarke J. Edwards T. (2016). Challenging games help students learn: an empirical study on engagement, flow and immersion in game-based learning. *Comput. Human Behav.* 54 170–179. 10.1016/j.chb.2015.07.045

[B44] Hansen D. M. Larson R. W. Dworkin J. B. (2003). What adolescents learn in organized youth activities: a survey of self-reported developmental experiences. *J. Res. Adolesc.* 13 25–55. 10.1111/1532-7795.1301006

[B45] Harari Y. N. (2008). *The Ultimate Experience: Martial Revelations and the Making of Modern war Culture.* Houndmills: Palgrave-Macmillan, 1450–2000.

[B46] Harmison R. J. (2006). Peak performance in sport: identifying ideal performance states and developing athletes’ psychological skills. *Prof. Psychol.* 37 233–243. 10.1037/0735-7028.37.3.233

[B47] Hernández B. Martin A. M. Ruiz C. Hidalgo M. D. (2010). The role of place identity and place attachment in breaking environmental protection laws. *J. Environ. Psychol.* 30 281–288. 10.1016/j.jenvp.2010.01.009

[B48] Huta V. (2012). Linking peoples’ pursuit of eudaimonia and hedonia with characteristics of their parents: parenting styles, verbally endorsed values, and role modeling. *J. Happiness Stud.* 13 47–61. 10.1007/s10902-011-9249-7

[B49] Irwin H. (1985). *Flight of Mind: A Psychological Study of the Out-of-Body Experience.* Metuchen, NJ: Scarecrow Press.

[B50] Jackson S. A. Marsh H. W. (1996). Development and validation of a scale to measure optimal experience: the flow state scale. *J. Sport Exerc. Psychol.* 18 17–35. 10.1123/jsep.18.1.17

[B51] Jorgensen B. S. Stedman R. C. (2001). Sense of place as an attitude: lakeshore owners attitudes toward their properties. *J. Environ. Psychol.* 21 233–248. 10.1006/jevp.2001.0226

[B52] Kowal J. Fortier M. S. (1999). Motivational determinants of flow: contributions from self-determination theory. *J. Soc. Psychol.* 139 355–368. 10.1080/00224549909598391

[B53] Kyle G. Bricker K. Graefe A. Wickham T. (2004a). An examination of recreationists’ relationships with activities and settings. *Leis. Sci.* 26 123–142. 10.1080/01490400490432019

[B54] Kyle G. Graefe A. Manning R. Bacon J. (2003). An examination of the relationship between leisure activity involvement and place attachment among hikers along the Appalachian trail. *J. Leis. Res.* 35 249–273.

[B55] Kyle G. Graefe A. Manning R. Bacon J. (2004b). Effect of activity involvement and place attachment on recreationists’ perceptions of setting density. *J. Leis. Res.* 36 209–231.

[B56] Kyle G. Graefe A. Manning R. Bacon J. (2004c). Predictors of behavioral loyalty among hikers along the appalachian trail. *Leis. Sci.* 26 99–118. 10.1080/01490400490272675

[B57] Lee T. H. Shen Y. L. (2013). The influence of leisure involvement and place attachment on destination loyalty: evidence from recreationists walking their dogs in urban parks. *J. Environ. Psychol.* 33 76–85. 10.1016/j.jenvp.2012.11.002

[B58] Mao Y. Roberts S. Bonaiuto M. (2016a). “Optimal experience and optimal identity: a multinational examination at the personal identity level,” in *Flow Experience: Empirical Research and Applications*, eds Harmat L. Andersen F. Ullén F. Wright J. (Switzerland: Springer), 289–308. 10.1007/978-3-319-28634-1_18

[B59] Mao Y. Roberts S. Pagliaro S. Csikszentmihalyi M. Bonaiuto M. (2016b). Optimal experience and optimal identity: a multinational study of the associations between flow and social identity. *Front. Psychol.* 7:67 10.3389/fpsyg.2016.00067 PMC4760053 26924995

[B60] Martin J. Cutler K. (2002). An exploratory study of flow and motivation in theatre actors. *J. Appl. Sport Psychol.* 14 344–352. 10.1080/10413200290103608

[B61] Maslow A. H. (1970). *Religions, Value, and Peak Experiences.* New York, NY: Penguin Books.

[B62] Massimini F. Csikszentmihalyi M. Delle Fave A. (1988). “Flow and biocultural evolution,” in *Optimal experience: Psychological studies of Flow in Consciousness*, eds Csikszentmihalyi M. Csikszentmihalyi I. (New Yourk, NY: Cambridge University Press), 60–81.

[B63] Nakamura J. Csikszentmihalyi M. (2002). “The concept of flow,” in *Handbook of Positive Psychology*, eds Snyder C. R. Lopez S. J. (Oxford: Oxford University Press), 89–105.

[B64] Nakamura J. Csikszentmihalyi M. (2009). “Flow theory and research,” in *Handbook of Positive Psychology*, eds Snyder C. R. Lopez S. J. (Oxford: Oxford University Press), 195–206.

[B65] Nielsen K. Cleal B. (2010). Predicting flow at work: investigating the activities and job characteristics that predict flow states at work. *J. Occup. Health Psychol.* 15 180–190. 10.1037/a0018893 20364915

[B66] Norton D. L. (1976). *Personal Destinies: A Philosophy of Ethical Individualism.* Princeton, NJ: Princeton University Press.

[B67] OECD (2015). *How’s Life? 2015: Measuring Well-Being.* Paris: OECD Publishing.

[B68] Prezza M. Amici M. Roberti T. Tedeschi G. (2001). Sense of community referred to the whole town: its relations with neighboring, loneliness, life satisfaction and area of residence. *J. Community Psychol.* 29 29–52. 10.1002/1520-6629(200101)29:1<29::AID-JCOP3>3.0.CO;2-C

[B69] Prezza M. Costantini S. Chiarolanza V. Di Marco S. (1999). La scalaitaliana del senso di co- munitaÌ. (*the Italian scale of sense of community)*. *Psicol. Salute* 3/4 135–159.

[B70] Proshansky H. M. (1978). The city and self identity. *Environ. Behav.* 10 147–169. 10.1177/0013916578102002

[B71] Proshansky H. M. Fabian A. K. (1987). “The development of place identity in the child,” in *Spaces for Children*, eds Weinstein C. S. David T. G. (New York, NY: Plenum), 21–40.

[B72] Proshansky H. M. Fabian A. K. Kaminoff R. (1983). Place identity: physical world socialization of the self. *J. Environ. Psychol.* 3 57–83. 10.1016/S0272-4944(83)80021-8

[B73] Ross W. D. (1949). *Aristotle*, 5th Edn London: Methuen.

[B74] Russell W. D. (2001). An examination of flow state occurrence in college athletes. *J. Sport Behav.* 24 83–107.

[B75] Ryff C. D. Singer B. H. (2006). Best news yet on the six-factor model of well-being. *Soc. Sci. Res.* 35 1103–1119. 10.1016/j.ssresearch.2006.01.002

[B76] Ryff C. D. Singer B. H. (2008). Know thyself and become what you are: a eudaimonic approach to psychological well-being. *J. Happiness Stud.* 9 13–39. 10.1007/s10902-006-9019-0

[B77] Schwartz S. J. (2006). Predicting identity consolidation from self-construction, eudaimonistic self-discovery, and agentic personality. *J. Adolesc.* 29 777–793. 10.1016/j.adolescence.2005.11.008 16426674

[B78] Schwartz S. J. Waterman A. S. (2006). Changing interests: a longitudinal study of intrinsic motivation for personally salient activities. *J. Res. Pers.* 40 1119–1136. 10.1016/j.jrp.2005.12.003

[B79] Seligman M. E. P. (2002). *Authentic Happiness: Using the New Positive Psychology to Realize Your Potential for Lasting Fulfilment.* New York: Free Press.

[B80] Sharp E. H. Coatsworth J. D. Darling N. Cumsille P. Ranieri S. (2007). Gender differences in the self-defining activities and identity experiences of adolescents and emerging adults. *J. Adolesc.* 30 251–269. 10.1016/j.adolescence.2006.02.006 16600358

[B81] Skadberg Y. X. Kimmel J. R. (2004). Visitors’ flow experience while browsing a web site: its measurement, contributing factors and consequences. *Comput. Human Behav.* 20 403–422. 10.1016/S0747-5632(03)00050-5

[B82] Stedman R. C. (1997). Where the north begins: cognitive maps, sense of place and place attachment *Paper presented at the Annual Meetings of the Rural Sociological Society*, Toronto, 13–17.

[B83] Tietze R. L. (2008). Jazz and american identity: case study of a college course. *Psychol. Aesthet. Creat. Arts* 2 245–255. 10.1037/a0010843

[B84] Twigger-Ross C. Bonaiuto M. Breakwell G. (2003). “Identity theories and environmental psychology,” in *Psychological Theories for Environmental Issues*, eds Bonnes M. Lee T. Bonaiuto M. (Aldershot: Ashgate, Ethnoscapes), 203–233.

[B85] Waterman A. S. (1984). Identity formation: discovery or creation? *J. Early Adolesc.* 4 329–341. 10.1177/0272431684044004

[B86] Waterman A. S. (1990a). Personal expressiveness: philosophical and psychological foundations. *J. Mind Behav.* 11 47–74.

[B87] Waterman A. S. (1990b). The relevance of Aristotle’s conception of eudaimonia for the psychological study of happiness. *Theor. Philos. Psychol.* 10 39–44. 10.1037/h0091489

[B88] Waterman A. S. (1992). “Identity as an aspect of optimal psychological functioning,” in *Identity Formation During Adolescence: Advances in Adolescent Development*, eds Adams G. R. Gullotta T. P. Montemayor R. (Newbury Park, CA: Sage), 50–72.

[B89] Waterman A. S. (1993a). “Finding something to do or someone to be: A Eudaimonist perspective on identity formation,” in *Discussions of ego Identity*, ed. Kroger J. (Hillsdale, NJ: Lawrence Erlbaum & Associates), 147–167.

[B90] Waterman A. S. (1993b). Two conceptions of happiness: contrasts of personal expressiveness (eudaimonia) and hedonic enjoyment. *J. Pers. Soc. Psychol.* 64 678–691. 10.1037/0022-3514.64.4.678

[B91] Waterman A. S. (2004). Finding someone to be: studies on the role of intrinsic motivation in identity formation. *Identity* 4 209–228. 10.1207/s1532706xid0403_1

[B92] Waterman A. S. (2005). When effort is enjoyed: two Studies of intrinsic motivation for personally salient activities. *Motiv. Emot.* 29 165–188. 10.1007/s11031-005-9440-4

[B93] Waterman A. S. (2007). On the importance of distinguishing hedonia and eudaimonia when contemplating the hedonic treadmill. *Am. Psychol.* 62 612–613. 10.1037/0003-066X62.6.612 17874913

[B94] Waterman A. S. Schwartz S. J. Conti R. (2008). The implications of two concept of happiness for the understanding of intrinsic motivation. *J. Happiness Stud.* 9 41–79. 10.1007/s10902-006-9020-7

[B95] Waterman A. S. Schwartz S. J. Goldbacher E. Green H. Miller C. Philip S. (2003). Predicting the subjective experience of intrinsic motivation: the roles of self-determination, the balance of challenges and skills, and self-realization values. *Pers. Soc. Psychol. Bull.* 29 1447–1458. 10.1177/0146167203256907 15189581

[B96] Williams D. R. Roggenbuck J. W. (1989). Measuring place attachment: some preliminary results *Paper presented at the Session on Outdoor Planning and Management, NRPA Symposium on Leisure Research*, San Antonio, Tx, 20–22.

[B97] Wöran B. Arnberger A. (2012). Exploring relationships between recreation specialization, restorative environments and mountain hikers’. *Flow Exp. Leis. Sci.* 34 95–114. 10.1080/01490400.2012.652502

